# Applications of the Clifford torus to material textures

**DOI:** 10.1107/S160057672400219X

**Published:** 2024-04-15

**Authors:** Marc De Graef

**Affiliations:** a Department of Materials Science and Engineering, Carnegie Mellon University, Pittsburgh, PA 15213-3890, USA; HPSTAR and Harbin Institute of Technology, People’s Republic of China

**Keywords:** orientation distribution functions, texture, symmetry, quaternions, Clifford torus

## Abstract

This paper introduces a new 2D representation of the orientation distribution function for an arbitrary material texture. The approach is based on the isometric square torus mapping of the Clifford torus, which allows for points on the unit quaternion hypersphere (each corresponding to a 3D orientation) to be represented in a periodic 2D square map. The combination of three such orthogonal mappings into a single RGB (red–green–blue) image provides a compact periodic representation of any set of orientations. Square torus representations of five different orientation sampling methods are compared and analyzed in terms of the Riesz *s* energies that quantify the uniformity of the samplings. The effect of crystallographic symmetry on the square torus map is analyzed in terms of the Rodrigues fundamental zones for the rotational symmetry groups. The paper concludes with example representations of important texture components in cubic and hexagonal materials. The new RGB representation provides a convenient and compact way of generating training data for the automated analysis of material textures by means of neural networks.

## Abstract 2

A new 2D representation of 3D material textures based on the Clifford torus is presented.

## 1. Introduction

The texture of a polycrystalline material is typically described by an orientation distribution function (ODF), which represents the volume fraction of the sample with a particular orientation with respect to an external reference frame. In the materials field, this is usually the sample frame described by the RD–TD–ND directions (reference, transverse and normal directions, respectively), whereas the geological community typically selects a sample reference frame associated with the growth history of the sample. Traditionally, the orientations are parameterized by means of three Euler angles; in the Bunge Euler convention, those angles are represented by the triplet (φ_1_, Φ, φ_2_) corresponding to rotations around the Cartesian *z*, *x*′ and *z* *′′* axes, respectively. There are several other frequently used rotation representations, including the set of neo-Eulerian representations (Rodrigues–Frank, homochoric or 3D stereographic vectors) and the quaternion representation; for details of each of these we refer the interested reader to Morawiec (2004 ▸). Details of the conversions between the representations can be found in the work of Rowenhorst *et al.* (2015 ▸).

Given the 3D nature of orientation space, the human brain’s ability to interpret 3D structures correctly, and the widespread availability of high-end computer graphics, several new orientation visualization techniques have been proposed in recent years (Berestova *et al.*, 2018 ▸; Krakow *et al.*, 2017 ▸; Callahan *et al.*, 2017*a* ▸,*b* ▸). In these articles, several orientation representations, in particular the neo-Eulerian representations, are combined with graphical rendering software to produce 3D visualizations of textures as point clouds and clusters, density functions, and emission maps, either displaying the full orientation space or applying restrictions to the Rodrigues fundamental zone for textures and to the disorientation fundamental zone for multi-phase textures (Callahan *et al.*, 2017*b* ▸).

Despite the elegance and widespread availability of these 3D renderings (*e.g.* in *MTEX*; Bachmann *et al.*, 2010 ▸), they are typically restricted to interactive environments where the user can manipulate the viewing direction, zoom in on particular regions of orientation space or change the orientation representation mode, *e.g.* from Rodrigues–Frank vectors to homochoric or 3D stereographic vectors. Furthermore, none of these 3D visualizations lend themselves to being analyzed with machine learning techniques. The main goal of this paper is to propose a 2D visualization of a texture that can be formatted as a periodic RGB (red–green–blue) color image and is hence suitable as training data for a classification neural network to recognize texture components and/or texture fibers automatically.

From a numerical point of view, the quaternion representation provides an efficient and powerful way of working with 3D rotations, for instance in combining rotations or finding a geodesic path between two different orientations (Hanson, 2006 ▸). In the following section, we will thus start from the three-sphere, 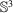 , *i.e.* the unit quaternion sphere, to derive a 2D representation of 3D orientation space that can potentially be used for the training of neural networks.

## 2. Mathematical background

### 2.1. Definitions

Following the original description in Section 4.4 of Alexa (2022 ▸), we define the Clifford torus, also known as the Euclidean two-torus, as the Cartesian product of two unit circles which results in an object embedded in 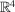 . Consider the unit circle 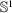 parameterized by the angle θ and defined as the set of all points of the form 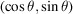 with 

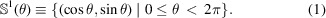

Scaling the circle to a radius of ρ and taking the Cartesian product of two such circles results in the Clifford torus, 

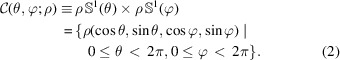

For the special choice 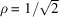 , the Clifford torus 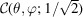 becomes a sub-manifold of the unit three-sphere, 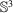 , because the norm of each point on the torus is equal to 1. Putting 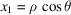 and 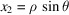 , we have 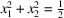 ; similarly, for 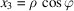 and 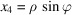 , we have 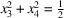 , so that 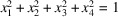 .

The Clifford torus has the special property that it is flat, *i.e.* there exists an isometry from the torus to a 2D square with periodic boundaries; the edges of the square have length 2π and cover the interval [−π, π]. The isometric mapping, which can be shown to have a unit Jacobian, consists of taking the ratios

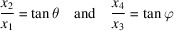

and inverting the relations to the coordinates (*X*, *Y*) = (θ, φ) in the square, 

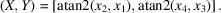

Here we use the traditional numerical two-argument version of the arctangent function 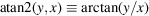 which produces angles in the range [−π, π].

In materials texture analysis, it is common practice to perform computations involving 3D orientations by means of unit quaternions, 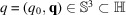 , with *q*_0_ the scalar part and **q** the vector part of the quaternion. Since unit quaternions reside on 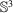 and the Clifford torus is a sub-manifold of the three-sphere, the following question arises naturally: *What role can the Clifford torus play in the description of sets of 3D orientations/rotations and, thus, the description of 3D materials textures?*

### 2.2. Projection of unit quaternions onto the Clifford torus

For a unit quaternion *q* with components (*q*_0_, *q*_1_, *q*_2_, *q*_3_), the projection on the Clifford torus 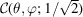 results in the point **x** with coordinates

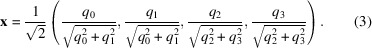

The projected coordinates in the square torus are then readily shown to be given by

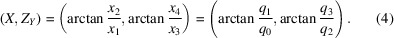

The reason for the use of the symbol *Z*_*Y*_ will become clear below. For a 3D rotation with unit rotation axis 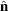 and rotation angle ω, the unit quaternion is given by

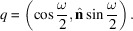

Substitution of the quaternion components *q*_*i*_ into equation (4) then results in

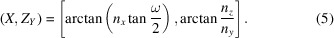

This can be converted to the Rodrigues–Frank representation by noting that **R** = 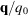 = 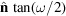 , so that 

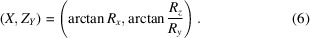

The notation *Z*_*Y*_ reflects the fact that the ratio *R*_*z*_/*R*_*y*_ is used to determine the second square torus coordinate. Note that this relation is not invertible, since there are only two degrees of freedom on the square torus and 3D rotations require three degrees of freedom. The representation in terms of the Rodrigues–Frank vector **R** suggests that the loss of information during the projection onto the square torus occurs due to the ratio of the *R*_*z*_ and *R*_*y*_ components in the *Z*_*Y*_ coordinate. This, in turn, suggests that two other projections can be defined by cyclic permutation of the vector components (*q*_1_, *q*_2_, *q*_3_) of the quaternion in equation (3); starting from this equation, we can derive two additional square torus projections via the relations 

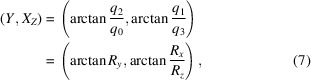




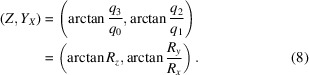

We can think of the three coordinate pairs as three different isometric projections of an orientation onto three orthogonal square tori. We will label the square tori by their coordinate symbols; when no coordinate label is present, the (*X*, *Z*_*Y*_) projection will be assumed. In terms of the Rodrigues–Frank vector components, the cyclic permutations correspond to 120° rotations about the principal diagonal axis of the Rodrigues reference frame.

**Figure 1:**
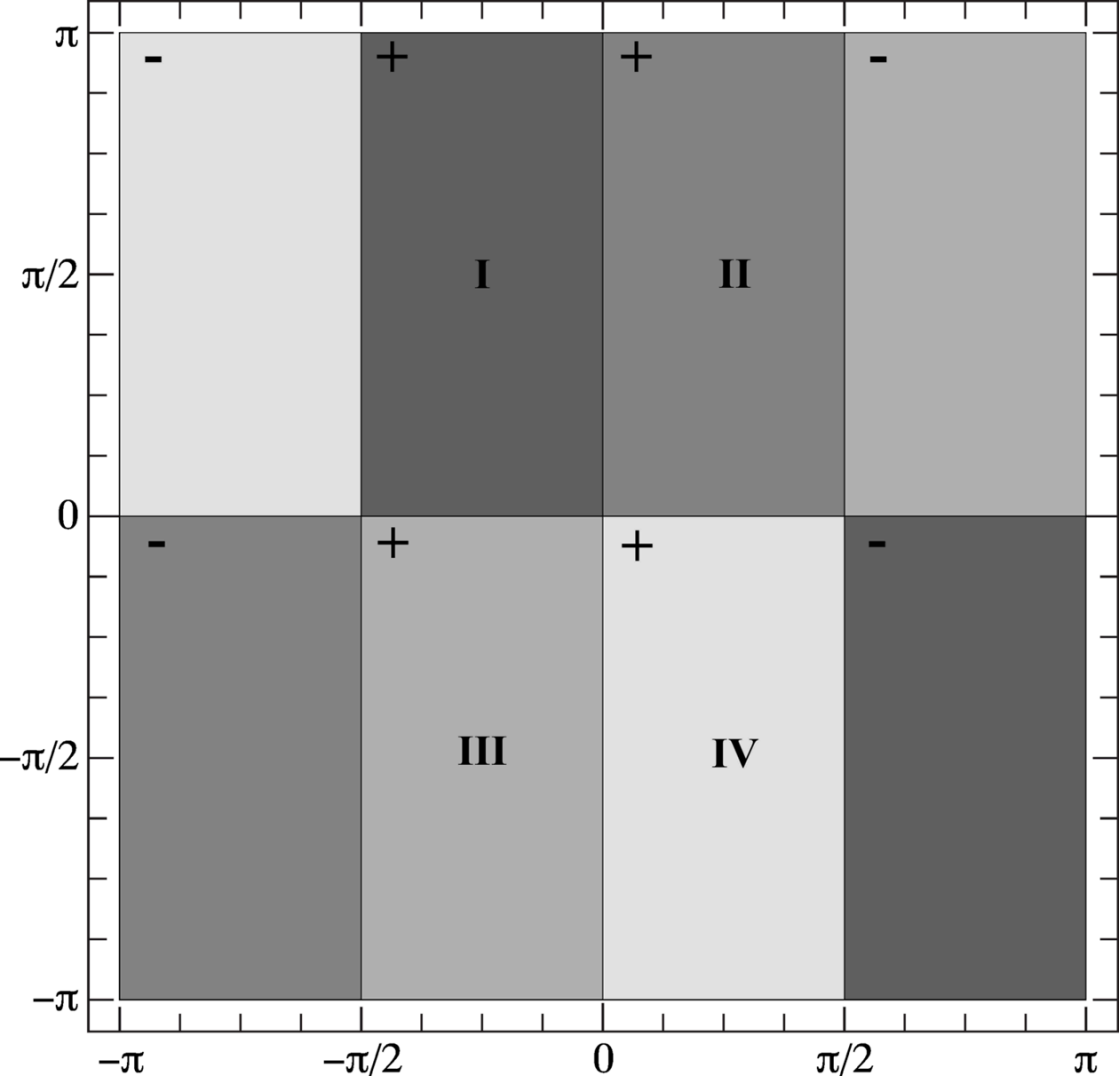
A square torus diagram, delineating regions with different sign combinations for the unit quaternion components. Rectangular regions with identical gray shading are translationally identical but have opposite sign for all quaternion components. Periodic boundary conditions apply in both horizontal and vertical directions.

Fig. 1 ▸ shows the square torus (*X*, *Z*_*Y*_) in the range [−π, π] along both horizontal and vertical axes; the left and right vertical edges connect to each other, as do the top and bottom edges. The shaded areas correspond to different sign combinations of the quaternion components, with regions I–IV corresponding to quaternions with a positive scalar part (the default convention for 3D rotations) and a negative scalar part for the outer regions. Rectangles with the same gray level are exact copies of each other due to the double-cover nature of 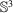 .

### 2.3. Relation between the square torus map and the Euler angle representation

The (*Z*, *Y*_*X*_) square torus map is related to a projection of Euler space along the Φ axis. The transformation relations from the Rodrigues vector components to the Bunge Euler angles are given by (Callahan *et al.*, 2017*a* ▸)

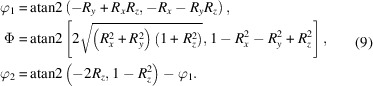

For the (*Z*, *Y*_*X*_) map, we have 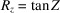 and 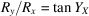 and, using the sum and difference formulas for the tangent function, it is easy to show that

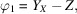




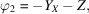

so that 

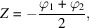




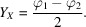

This means that the (*Z*, *Y*_*X*_) square torus map is identical to a projection of Euler space along the Φ axis followed by a 45° rotation, bringing the φ_1_ = φ_2_ diagonal parallel to the *Y*_*X*_ axis of the square torus map. The two other maps, (*X*, *Z*_*Y*_) and (*Y*, *X*_*Z*_), do not appear to have simple interpretations in terms of linear projections through Euler space; they are more complicated nonlinear projections.

### 2.4. Zone-plate function representation

For a given set 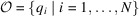 of *N* unit quaternions, one can represent each quaternion by a narrow symmetric normalized 2D Gaussian function at the position (*X*, *Z*_*Y*_) and add all Gaussians together to obtain a 2D ‘intensity’ landscape 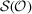 representing the set 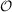 . Several versions of this landscape can be generated:

(i) 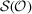 . All orientations in the set are represented visually, without application of symmetry or conversion to quaternions with a positive scalar part.

(ii) 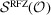 . All orientations are reduced to the Rodrigues fundamental zone (RFZ) for the rotational point group corresponding to the crystal structure (this implies that they have a positive scalar part).

(iii) 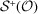 or 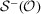 . All symmetrically equivalent orientations are computed but only those with either a positive or a negative scalar part are visualized on the square torus.

(iv) 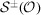 . All symmetrically equivalent orientations with both positive and negative scalar quaternion parts are represented.

For each of these cases, one can apply a zone plate to the square torus, *i.e.* the intensity at each point is multiplied by a modulation function with spatially varying frequency content (Öztireli, 2020 ▸). For orientation sets that are supposed to be uniform in *SO*(3), the application of a zone plate sometimes facilitates the interpretation of the intensity distribution and makes it easier to spot non-uniformities. When a zone-plate function is applied, we represent this by a subscript on the intensity landscape, *e.g.* 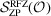 or 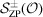 . In the following section we will explore what these representations look like for uniform samples of orientations, as well as for a number of well known textures. It should be noted that application of a zone-plate function in general destroys the equivalence of regions with equal shading in Fig. 1 ▸.

**Figure 2:**
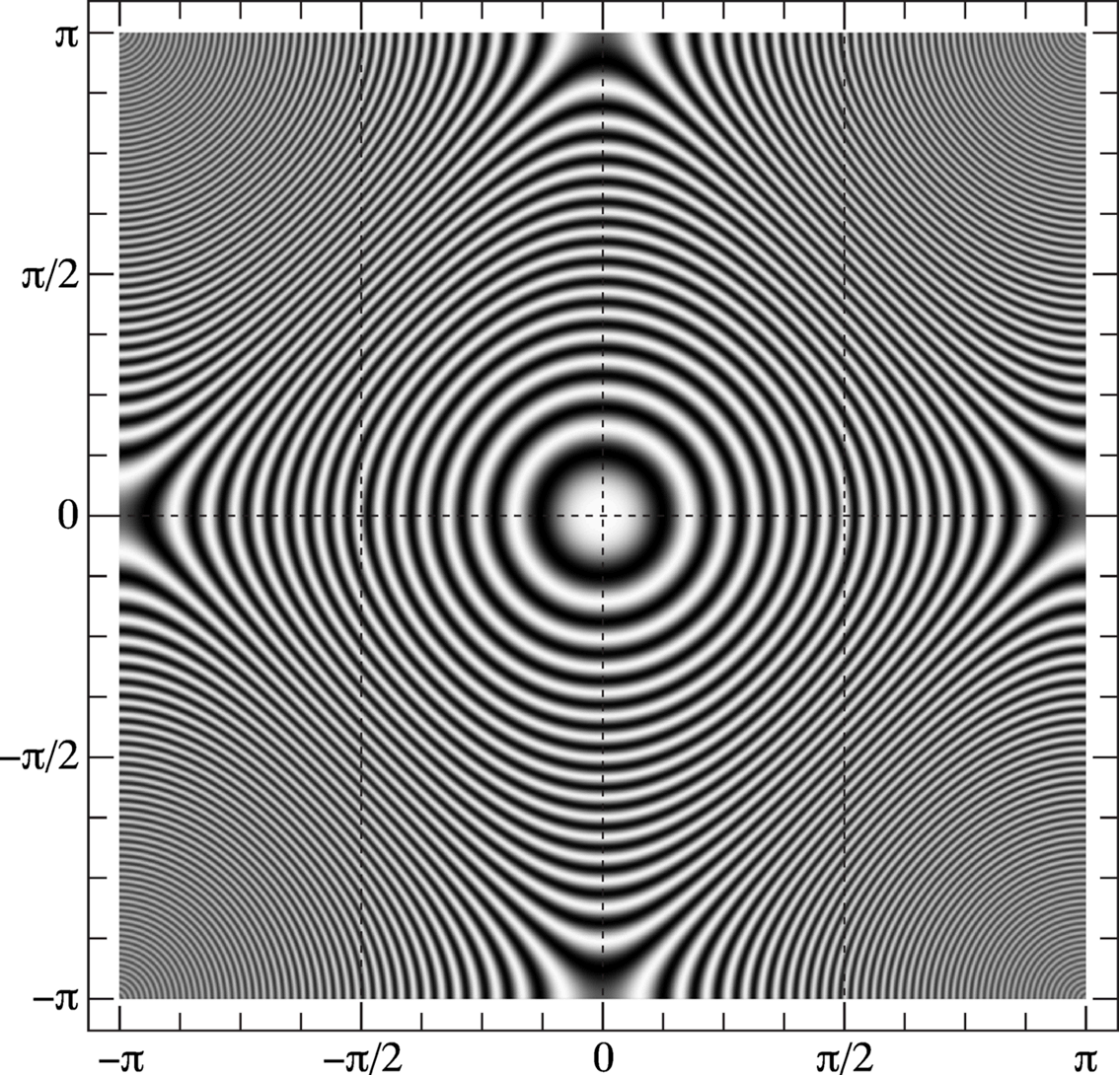
The zone-plate function [equation (10)] superimposed on the square torus diagram. Parameters are defined in the text.

Alexa (2022 ▸) suggested the following zone-plate function: 

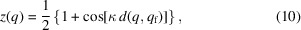

where κ is a constant that determines the number of oscillations of the zone-plate function in the interval [−π, π] and *d*(*p*, *q*) is the natural metric on 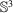 ,

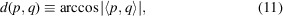

with 〈*p*, *q*〉 the standard dot product between two quaternions projected onto the Clifford torus. *q*_f_ is an arbitrary point on the torus, so that the zone-plate function uses the geodesic distance between *q* and *q*_f_ along the surface of the torus. In this paper, we select the reference point

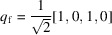

which is clearly located on the Clifford torus 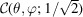 and causes *z*(*q*) to be symmetric with respect to the point (*X*, *Z*_*Y*_) = (0, 0), as shown in Fig. 2 ▸; in this figure, we have set κ = 40 and subdivided the interval [−π, π] into an equidistant grid of 1001 points along both axes.

## 3. Uniform and random samplings of *SO*(3)

In this section, we explore a number of different orientation sampling approaches and their representation on the square torus using a zone-plate function. The following sampling approaches are used to generate orientation sets:

(i) 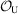 . Each quaternion is composed of four components, each uniformly sampled on the interval [−1, 1] using the Mersenne twister algorithm (Matsumoto & Nishimura, 1998 ▸), and the quaternion is subsequently normalized.

(ii) 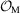 . Each unit quaternion is generated using the Marsaglia sampling approach (Marsaglia, 1972 ▸). Draw two uniform random numbers *x*_1_ and *y*_1_ from [−1, 1] until 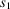 = 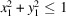 . Repeat for *x*_2_, *y*_2_ until 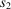 = 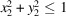 . Then replace *s*_2_ by 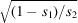 and form the unit quaternion *q* = [*x*_1_, *y*_1_, *x*_2_ *s*_2_, *y*_2_ *s*_2_].

(iii) 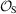 . In the Shoemake algorithm (Shoemake, 1992 ▸), three random numbers are generated using the Mersenne twister algorithm: *u*_1_ is selected uniformly from the interval [0, 1] and *u*_2_ and *u*_3_ are selected uniformly from the interval [0, 2π]. Setting *a* = 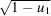 and *b* = 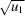 , the random unit quaternion is then generated as *q* = 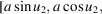 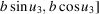 .

(iv) 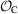 . Cubochoric sampling (Roşca *et al.*, 2014 ▸) is used to generate a uniform 3D grid of points inside a cube of edge length 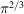 . Each of these points is then mapped using an equal-volume mapping onto the northern hemisphere of 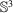 (the northern hemisphere corresponds to quaternions with positive scalar part), resulting in a uniform sampling of *SO*(3).

(v) 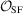 . Super-Fibonacci sampling (Alexa, 2022 ▸) is a relatively new sampling approach that relies on two irrational numbers (ϕ, ψ). One potential choice is related to the golden ratio (positive root of ϕ^2^ = ϕ + 1) and the super-golden ratio (positive root of ψ^3^ = ψ^2^ + 1). Another choice, producing a uniform set of quaternions with a lower dispersion, sets 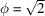 and ψ as the positive root of ψ^4^ = ψ + 4 → ψ = 1.53375117. To generate *N* uniformly distributed quaternions, the algorithm is as follows: for *i* ∈ [0, …, *N* − 1], set *s* = *i* + 1/2, *t* = *s*/*n*, *d* = 2π*s*, 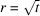 , 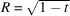 , α = *d*/ϕ and β = *d*/ψ. The quaternion *q*_*i*_ is then formed as 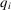 = 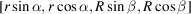 .

**Figure 3:**
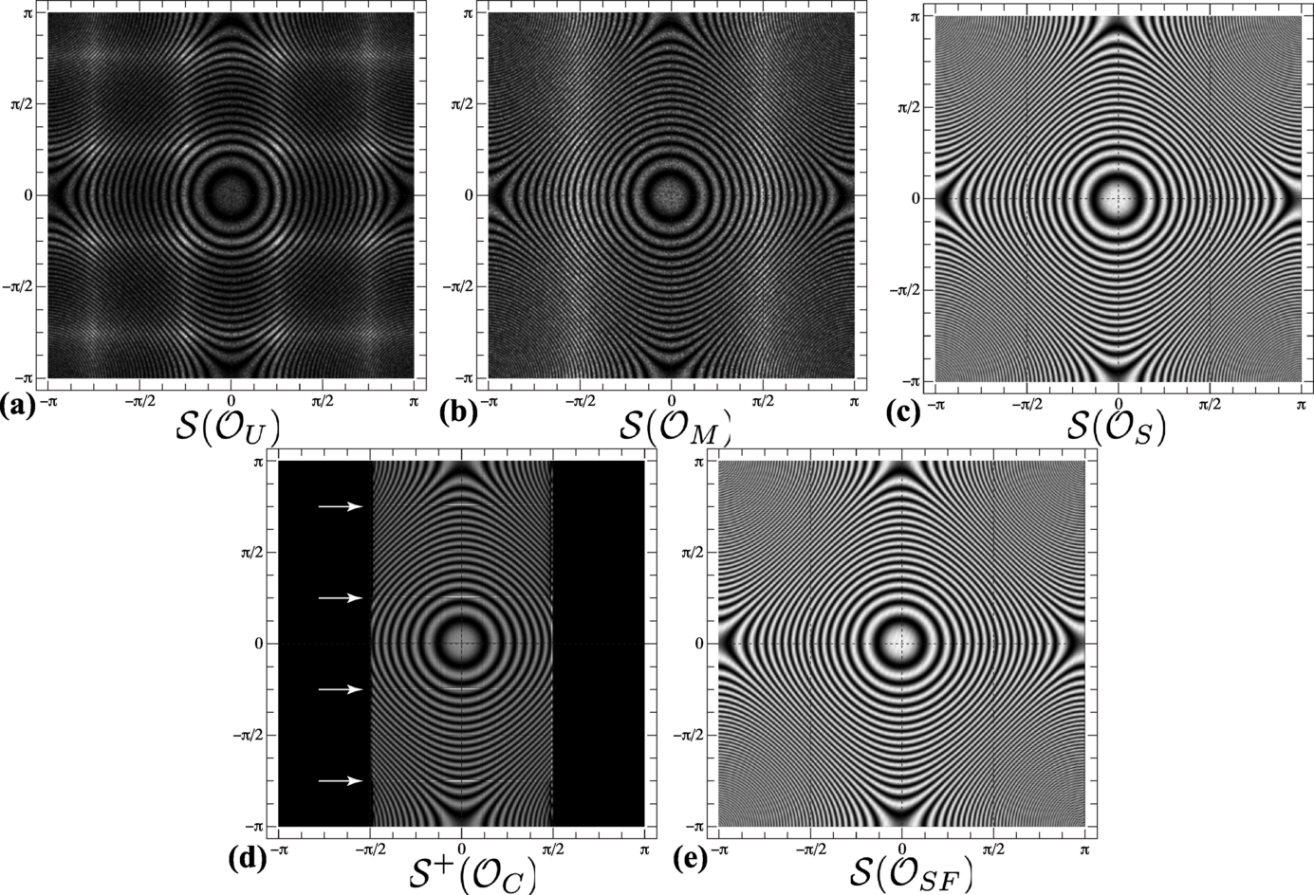
Zone-plate representations of samples of 10^6^ orientations using (*a*) uniform sampling 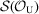 , (*b*) Marsaglia sampling 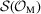 , (*c*) Shoemake sampling 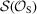 , (*d*) cubochoric sampling (northern hemisphere of **S** ^3^ only) 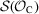 and (*e*) super-Fibonacci sampling 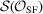 .

For each of these five sampling approaches, an orientation set of 10^6^ samples was generated and represented using the zone-plate function approach. For each orientation in the set, the quaternion was projected onto the Clifford torus and, at the corresponding point (*X*, *Y*) in the square torus, a narrow unit-amplitude 2D Gaussian kernel was added to the intensity plot and multiplied by the zone-plate function value for that point. The resulting intensity plots are shown in Fig. 3 ▸. Due to the discrete grid nature of cubochoric sampling, the number of sampling points for the set 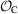 in general cannot be set arbitrarily; however, in the absence of crystal symmetry, a sampling of the cubochoric grid with 100 equidistant points on the side results in a sampling of orientation space with precisely 10^6^ points.

For the set 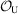 , the zone-plate function 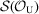 shown in Fig. 3 ▸(*a*) shows a regular pattern of excess intensities corresponding to (*X*, *Y*) values close to π/4 and 3π/4, indicating that the uniform sampling approach based on the Mersenne twister does not produce a uniform orientation sampling. For the Marsaglia generator, the function 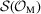 shown in Fig. 3 ▸(*b*) shows a better uniformity than the uniform sampling approach, but there is an excess intensity for orientations near *X* = ±π/2. The Shoemake algorithm produces a smoothly varying zone-plate representation [Fig. 3 ▸(*c*)], indicating that the sampling is uniform. The cubochoric sampling approach shows a relatively smooth zone-plate function 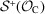 [Fig. 3 ▸(*d*)], but there are highly localized excess intensities (arrowed) for *Y* = ±π/4 and *Y* = ±3π/4. These are probably due to the fact that the cubochoric sampling algorithm relies on sampling of six pyramidal volumes that together make up the cube [see Fig. 1 in the report by Roşca *et al.* (2014 ▸)]; where the pyramids meet (along the body diagonals of the cube) the sampling is apparently not as uniform as elsewhere inside the cube. However, the overall zone plate for the cubochoric sampling approach is significantly smoother than that for the uniform and Marsaglia sampling methods, indicating that the orientation set 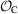 represents a more uniform sampling of *SO*(3). Finally, the zone-plate function 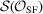 in Fig. 3 ▸(*e*) shows that the super-Fibonacci sampling approach produces a smooth intensity profile with no obvious non-uniformities.

The uniformity of a sampling of points on the sphere 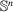 can be quantified using the concept of *Riesz energy* (Hardin & Saff, 2004 ▸). For a set 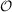 of *N* orientations represented by unit quaternions, the Riesz energy 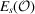 is defined as

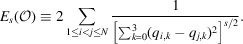

This expression can be interpreted as a generalized Coulomb energy for a collection of points on the sphere 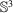 and reduces to the standard Coulomb energy for *s* = 1. The sum covers the entire three-sphere, not just the northern hemisphere 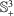 . The optimal Riesz energies for uniform coverage of any hypersphere are well known (Hardin & Saff, 2004 ▸); the values for the three-sphere for an orientation set with *N* elements are given by

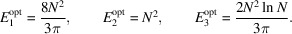




Table 1 ▸ lists the optimal values for an orientation data set of 2 × 10^6^ unit quaternions (counting *q* and −*q* as distinct points) along with the relative values 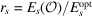 for each of the orientation sets of Fig. 3 ▸; for an optimal sampling, all three ratios should be equal to unity. Both uniform and Marsaglia sampling have ratios that are significantly different from unity, in particular the *r*_3_ value, which is more sensitive than the other two in terms of local sampling non-uniformities. The Shoemake algorithm produces *r*_1_ and *r*_2_ ratios that are very close to unity, but has an *r*_3_ value nearly twice the optimal value; this indicates that globally this sampling has excellent uniformity but on a local scale the sampling is not as optimal as the cubochoric and super-Fibonacci sampling approaches. The latter two are comparable in their *r*_*i*_ values, which are all relatively close to unity, indicating that there is no significant over-sampling of sub-regions of orientation space. The visualization of the orientation data sets in terms of the zone-plate function is qualitatively consistent with the Riesz energy ratios, so the zone-plate images can be used as a visual substitute for the more accurate computation of the Riesz energies.

## 4. Material textures and the square torus representation

### 4.1. Fundamental zone representations

In this section we review how orientation information restricted to an RFZ is represented in the square torus. The example orientation data sets used in Sections 4.2 and 4.3 are taken from the supplementary material of Callahan *et al.* (2017*b* ▸). In the context of material textures, the two additional square tori (*Y*, *X*_*Z*_) and (*Z*, *Y*_*X*_) become relevant since they project the orientation data along different directions. One can think of a texture representation as an intensity distribution on three adjacent faces of a cube with edge length 2π, or as a periodic RGB color image.

#### 4.1.1. Cyclic point-group symmetry

For the cyclic rotational point groups **2** (*C*_2_), **3** (*C*_3_), **4** (*C*_4_) and **6** (*C*_6_), the RFZ corresponds to the region between two parallel planes normal to the *R*_*z*_ axis [for the monoclinic point group **2** (*C*_2_), with *b* as the unique axis, the planes are normal to the *R*_*y*_ axis] at a distance of 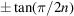 from the origin, where *n* is the order of the rotation axis. Points inside this region are of the form

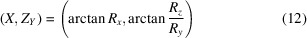

with 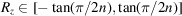 for *n* = 3, 4 and 6, and of the form 

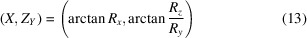

with 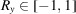 for *n* = 2. Producing a uniform sampling of these RFZs results in non-uniform distributions in the square torus because there are more points far away from the origin than nearby.

**Figure 4:**
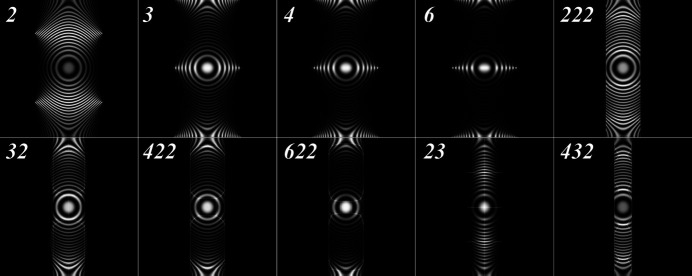
Zone-plate representations 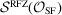 of uniform samplings of the Rodrigues fundamental zone for the rotational point groups. Thin white lines represent the projections of the edges of the RFZ onto the square torus, as shown more clearly in Fig. 5.

The zone-plate functions for the cyclic rotational point groups are shown in the top row in Fig. 4 ▸. The orientation sets were generated by the super-Fibonacci algorithm and subsequently reduced to the Rodrigues fundamental zone, *i.e.* the intensity plots are of the type 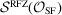 . Note that for **2** (*C*_2_), the denser (brighter) regions are shifted up by half a unit due to the selection of the *b* axis as the unique monoclinic axis. Along the horizontal axis, orientations span the range [−π/2, π/2], whereas in the vertical direction the entire range [−π, π] is used. As the order of the rotation axis increases, the intensity becomes more focused near the points (±π/2, 0).

#### 4.1.2. Dihedral, tetrahedral and octahedral point-group symmetry

**Figure 5:**
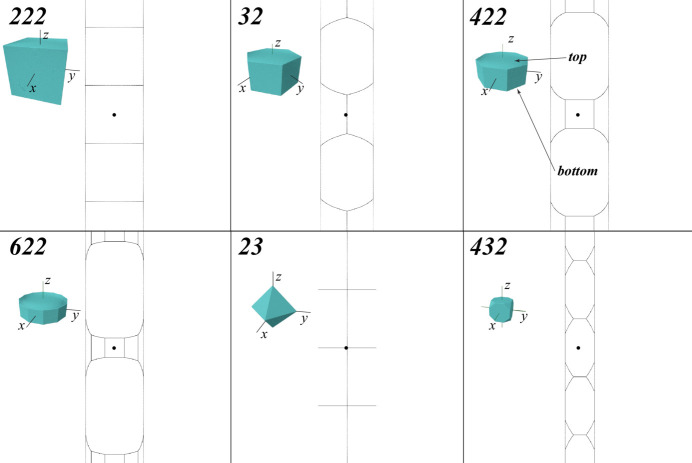
Outlines of the projections of the finite Rodrigues fundamental zones (shown as volume renders in the insets) for the rotational point groups indicated in the top left. Each square has coordinate ranges [−π, π] along both axes. The RFZ renderings use a common length scale.

For the dihedral point groups **222** (*D*_2_), **32** (*D*_3_), **422** (*D*_4_) and **622** (*D*_6_) and for the two cubic groups **23** (*T*) and **432** (*O*), the RFZs are finite and bounded by planar facets. Fig. 5 ▸ shows the projections of the RFZ edges onto the square torus along with a volume rendering of the RFZ; all inset images have the same scale and the *R*_*z*_ axis is vertical in all cases. The corresponding zone plates 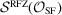 for these point groups are shown in the bottom row of Fig. 4 ▸.

A few general trends can be observed in the zone plates for the four dihedral groups:

(i) The projected RFZ outlines in Fig. 5 ▸ have the same horizontal width and stretch across the entire vertical dimension for all four dihedral groups. One can think of the projected outline as a distorted 2D net corresponding to the polyhedral RFZ shape. For instance, for point group **422** (*D*_4_) the central square in the outline corresponds to the square face normal to the *x* axis in Rodrigues space and the entire polyhedron is ‘unfolded’ in the vertical (*z*) direction to produce the distorted octagons above and below (labeled ‘top’ and ‘bottom’ in Fig. 5 ▸). The other vertical facets give rise to the curved areas at the *Y* = 0 line as well as the top and bottom *Y* = ±π regions of the square torus. The RFZ facets perpendicular to the *y* axis are projected onto the vertical *X* = ±π/2 edges of the outline.

(ii) The zone-plate plots in Fig. 4 ▸ show that the intensity becomes more concentrated along the *Y* = 0 and *Y* = ±π lines as the rotational order increases. This is because the ‘thickness’ of the RFZ along the *z* axis (Fig. 5 ▸) decreases [recall that the top and bottom facets are at a distance 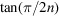 from the origin] and this results in fewer projected orientations near the *Y* = ±π/2 lines.

For the cubic rotational groups **23** (*T*) and **432** (*O*), the width of the zone plate corresponding to the RFZs is narrower than that for the dihedral groups. For the tetrahedral group **23** (*T*), the vertical edges of the region with non-zero intensity correspond to the single intersection points of the octahedral RFZ (Fig. 5 ▸) with the *y* axis; those orientations are completely degenerate in the square torus representation. For both cubic groups, the zone-plate intensity shown in Fig. 4 ▸ reaches a maximum near the horizontal projections of the RFZ edges.

### 4.2. Basic texture-type representations

In this section, we use two basic texture components, the cube or {100}〈001〉 texture and the Goss texture {110}〈001〉, to demonstrate how these orientation sets are represented using the square torus and/or the zone-plate intensity map. Synthetic cube and Goss textures were generated using the *EMsampleRFZ* program which is part of the *EMsoft* open source package for electron scattering simulations (Singh *et al.*, 2017 ▸). Each orientation set contains 1 000 000 unique orientations clustered around the respective texture component mean orientations, *i.e.* the origin of Rodrigues space for the cube texture and the point 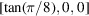 for the Goss texture {*i.e.* texture component (100)[011]}. The orientations were generated using von Mises–Fisher sampling with half-widths of 5° for the Goss texture and 10° for the cube texture.

**Figure 6:**
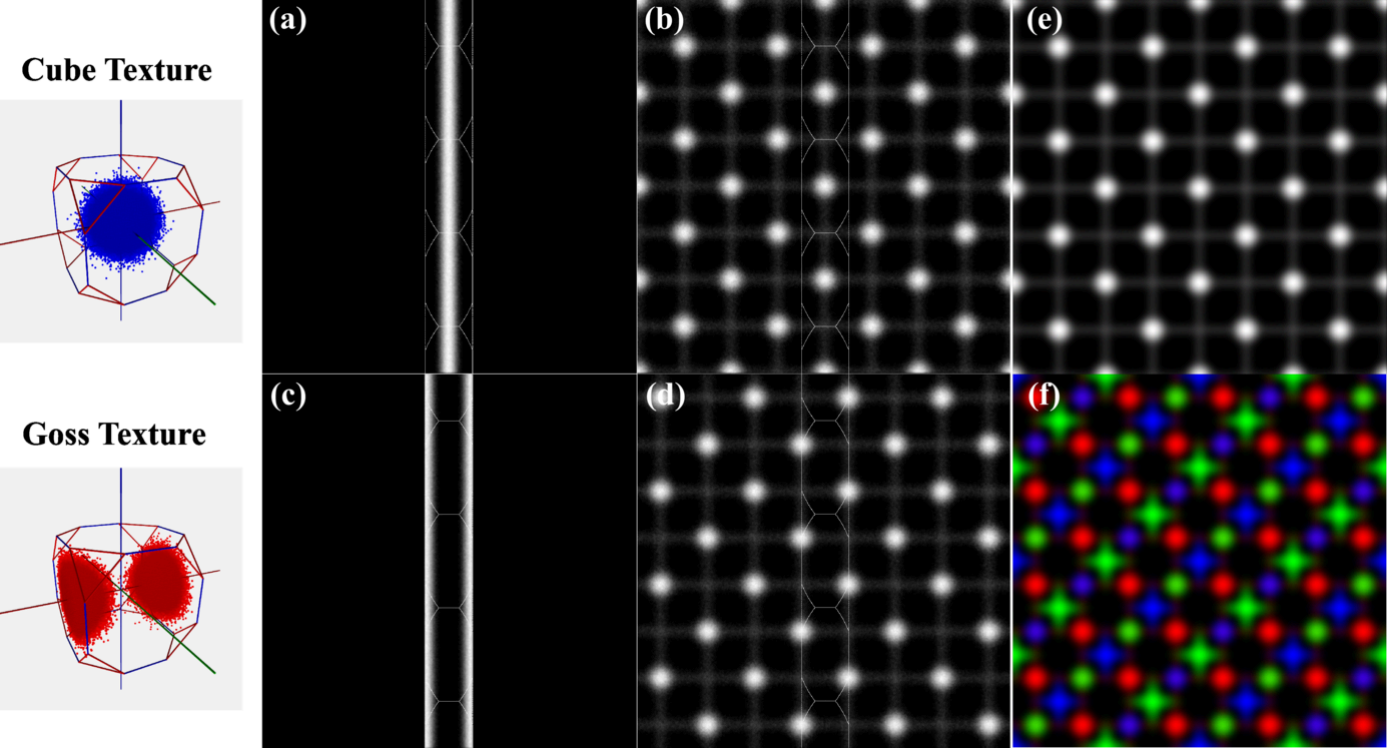
Cube (top row) and Goss (bottom row) textures represented on the square torus. The left-most column shows the two textures represented as point clusters in the cubic Rodrigues fundamental zone. The second column shows the square torus projections for the RFZ only, whereas in the third column the cubic symmetry operators, including the equivalence of *q* and −*q*, have been applied to the orientation data set. In the final column, three square torus map projections are combined into an RGB image.

Fig. 6 ▸ shows the two orientation distributions represented as point clouds in the cubic RFZ (left-most column); note that the cluster spans the RFZ boundaries for the Goss texture. Figs. 6 ▸(*a*) and 6 ▸(*c*) show the square torus (ST) representations for both textures, 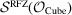 and 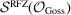 , along with the RFZ outlines. All orientations were reduced to the fundamental zone before being projected onto the square torus. In both cases, the representations contain vertical lines due to the ‘unfolding’ of the fundamental zone along the vertical direction of the ST map. In Figs. 6 ▸(*b*) and 6 ▸(*d*) the cubic rotational symmetry operations were applied to the orientation set (including the equivalence of quaternions *q* and −*q*) before projection; the resulting ST maps correspond to 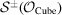 and 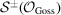 . Note that the Goss map is identical to the cube map, but is shifted diagonally by a vector (*X*, *Y*) = (π/8, −π/8).

Figs. 6 ▸(*e*) and 6 ▸(*f*) show RGB representations of the three square torus maps, with (*X*, *Z*_*Y*_) mapped onto the red channel, (*Y*, *X*_*Z*_) onto green and (*Z*, *Y*_*X*_) onto blue. For the cube texture in Fig. 6 ▸(*e*), the three maps are identical so that the RGB image becomes a grayscale image. For the Goss texture in Fig. 6 ▸(*f*), on the other hand, the three projections differ from each other and the resulting RGB image shows distinct clusters in each color.

**Figure 7:**
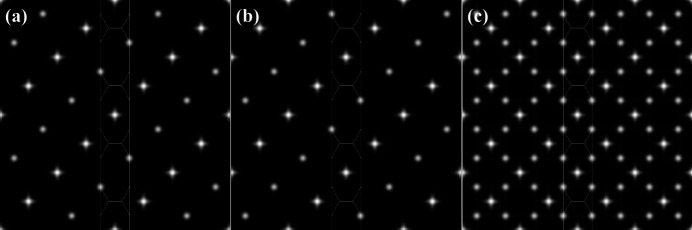
(*a*) 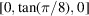 and (*b*) 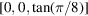 Goss textures represented on the square torus. An equally weighted mixture of the Goss texture along the three coordinate directions results in the superposition shown in panel (*c*). Each component corresponds to a Watson sample with 1 000 000 orientations and a concentration of κ = 262.8, corresponding to Δθ = 5°.

Because of the asymmetric way in which the *R*_*x*_ and (*R*_*y*_, *R*_*z*_) components of a Rodrigues vector contribute to the coordinates (*X*, *Y*) in the square torus [equation (6)], different symmetrically equivalent texture components will have different ST representations. Figs. 7 ▸(*a*) and 7 ▸(*b*) show the ST representations of the Goss texture components centered on the points 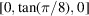 and 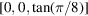 , respectively, and Fig. 7 ▸(*c*) shows the sum of all three Goss maps for equal weights.

**Figure 8:**
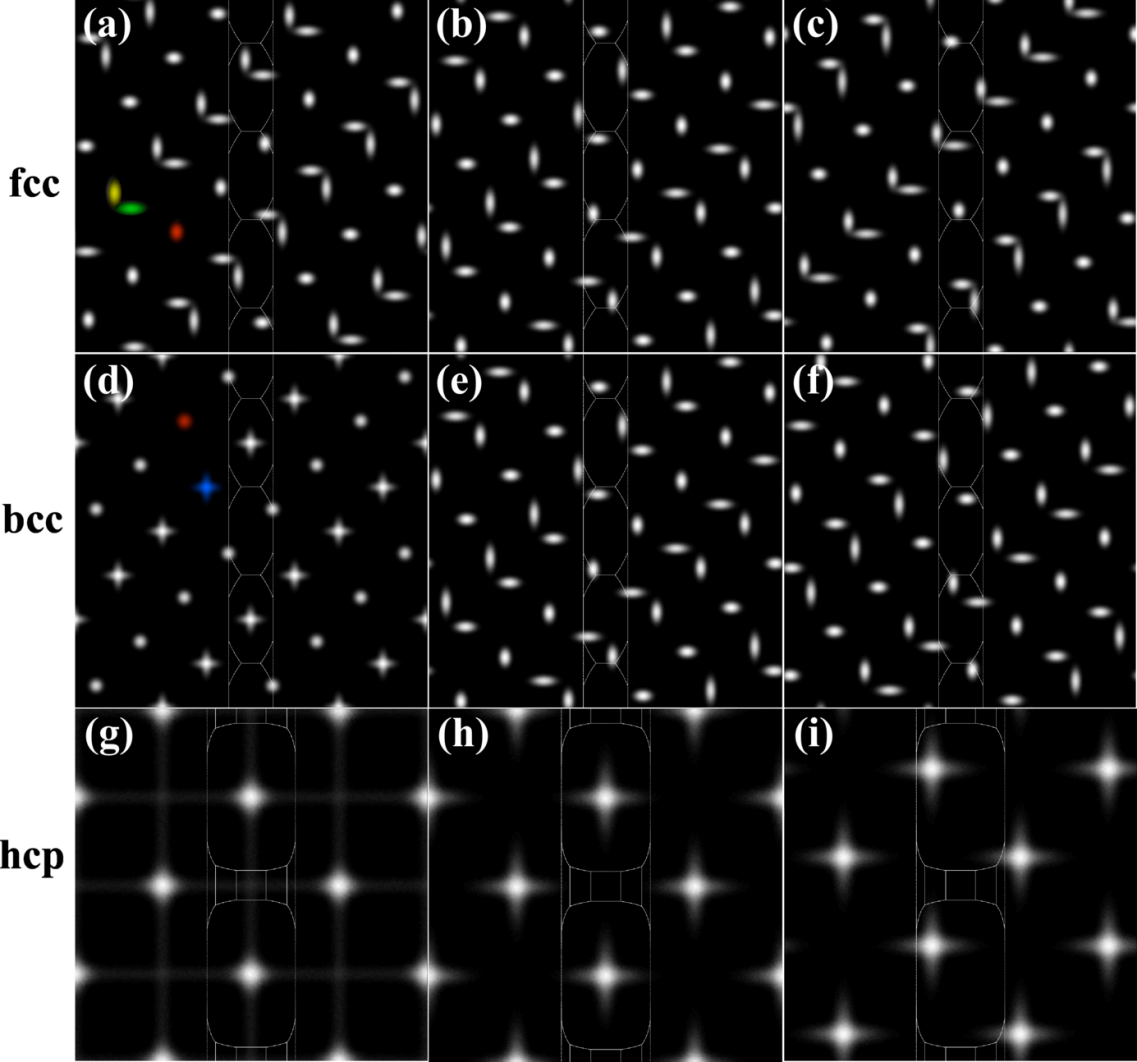
Example square torus representations of common texture components for the f.c.c., b.c.c. and h.c.p. crystal structures. F.c.c.: (*a*) 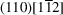 (brass), (*b*) 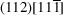 (copper), (*c*) 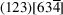 (S). B.c.c.: (*d*) (001)[110] (rotated cube), (*e*) 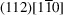 (inverse brass), (*f*) 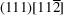 . H.c.p.: (*g*) (00.1)[10.0], (*h*) (00.1)[11.0], (*i*) (11.3)[10.0].

Fig. 8 ▸ shows the square torus representations for several common rolling texture components for face-centered cubic (f.c.c.), body-centered cubic (b.c.c.) and hexagonal close-packed (h.c.p.) crystal structures, as indicated in the figure caption. For cubic symmetry, both f.c.c. and b.c.c., the intensity distributions show three different basic components: a nearly circular peak [colored red in Fig. 8 ▸(*a*)] or a circular peak [red in Fig. 8 ▸(*d*)], a horizontally elongated peak (horizontal ellipse, green) and a vertically elongated peak (vertical ellipse, yellow). The texture components differ in the relative positioning of these three basic elements. In Fig. 8 ▸(*d*), the ellipses overlap, giving rise to a cross-like appearance (blue).

**Figure 9:**
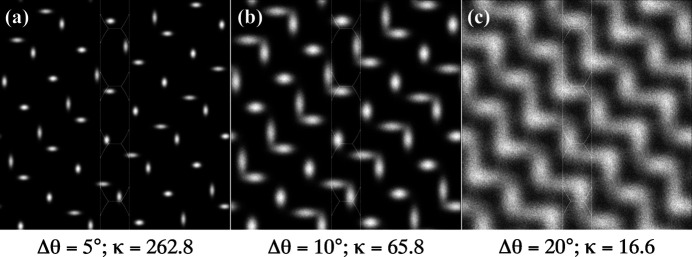
Square torus representation of the cubic 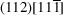 copper texture component as a function of the concentration parameter κ of the Watson distribution. Δθ is the corresponding angular spread of the distribution.

The size of the intensity peaks increases nonlinearly with the angular range Δθ (or, equivalently, the concentration parameter κ of the Watson distribution used to generate the samples). This is illustrated in Fig. 9 ▸, which shows the f.c.c. 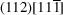 (copper) texture component for Δθ = 5°, 10° and 20°; the corresponding concentration values κ for the Watson distribution are shown below the intensity maps.

### 4.3. Experimental texture representations

**Figure 10:**
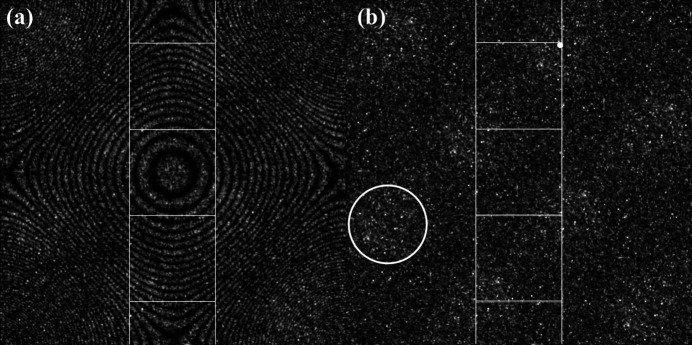
(*a*) Zone-plate and (*b*) square torus representations of the combined orientations from three EBSD scans (7 039 154 scan points in total) of a polycrystalline forsterite sample with random texture. The small white dot in the upper right portion of the RFZ outline represents the projection of the mean orientation quaternion μ for a Watson distribution fit to the orientation set (see text). The circled area in panel (*b*) highlights a cluster of higher intensity. (Data sets courtesy K. Marquardt, University of Oxford.)

In this section we consider several experimental data sets obtained using the electron backscatter diffraction (EBSD) technique. The first data set consists of three separate EBSD scans of a polycrystalline sample of the orthorhombic mineral forsterite (Mg_2_SiO_4_, space group No. 62, *Pbnm*, also known as olivine). The three data sets (courtesy of Dr K. Marquardt, University of Oxford, UK) were acquired at a beam energy of 20 keV and have a combined total of 7 039 154 sampling points covering an area of about 7 mm^2^ with a step size of 1 µm. The zone-plate and square torus representations of this orientation data set are shown in Figs. 10 ▸(*a*) and 10 ▸(*b*), respectively. The intensity distributions are very nearly uniform with a few higher-intensity clusters (one is circled in the square torus map), indicating that the texture of this sample is nearly random. There are eight equivalent higher-intensity cluster regions due to the order of the rotational point group (4) and the equivalence of *q* and −*q* (2).

To quantify the slight non-uniformity of this orientation set, a maximum likelihood estimation was performed of the mean orientation quaternion μ and concentration parameter κ for the Watson distribution on the three-sphere 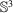 , under application of the rotational symmetry group **222** and the double-cover property; the details of the fitting algorithm are described by Chen *et al.* (2015 ▸). The fitted mean orientation is given by the unit quaternion, 

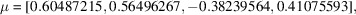

corresponding to the Rodrigues vector

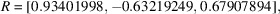

and square torus coordinates (*X*, *Y*) = (0.75129592, 2.3204532); this point is indicated by a small white dot in Fig. 10 ▸(*b*). This corresponds to a rotation of 105.56° around the [0.70946461, −0.48020196, 0.51581597] axis in the (RD, TD, ND) sample reference frame. The Watson concentration parameter is given by κ = 2.1 (or 58.4°), indicating a very weak and broad clustered texture. This is in agreement with a *T*-matrix analysis (Mardia & Jupp, 2009 ▸), which results in the eigenvalues λ_*i*_ = [0.613515, 0.142532, 0.126213, 0.117740]; the largest eigenvalue is only about four times larger than the others, which indicates a weakly clustered distribution.

For the synthetic orientation sets from Section 4.2 (and also in Fig. 9 ▸), the concentration parameters are one to two orders of magnitude larger than for the forsterite data set and the clustering around the mean orientation is clearly observed in the square torus representation. For the cube texture of Fig. 6 ▸(*a*), the eigenvalues of the *T*-matrix are λ_*i*_ = [0.983452, 0.005506, 0.005516, 0.005525]; the ratio of the first two eigenvalues is 178.6, indicating a strongly clustered texture, in agreement with the Watson distribution κ value of 91.2. These results indicate that simple visual observation of the square torus representation can reveal even very small non-uniformities in the orientation distribution function.

**Figure 11:**
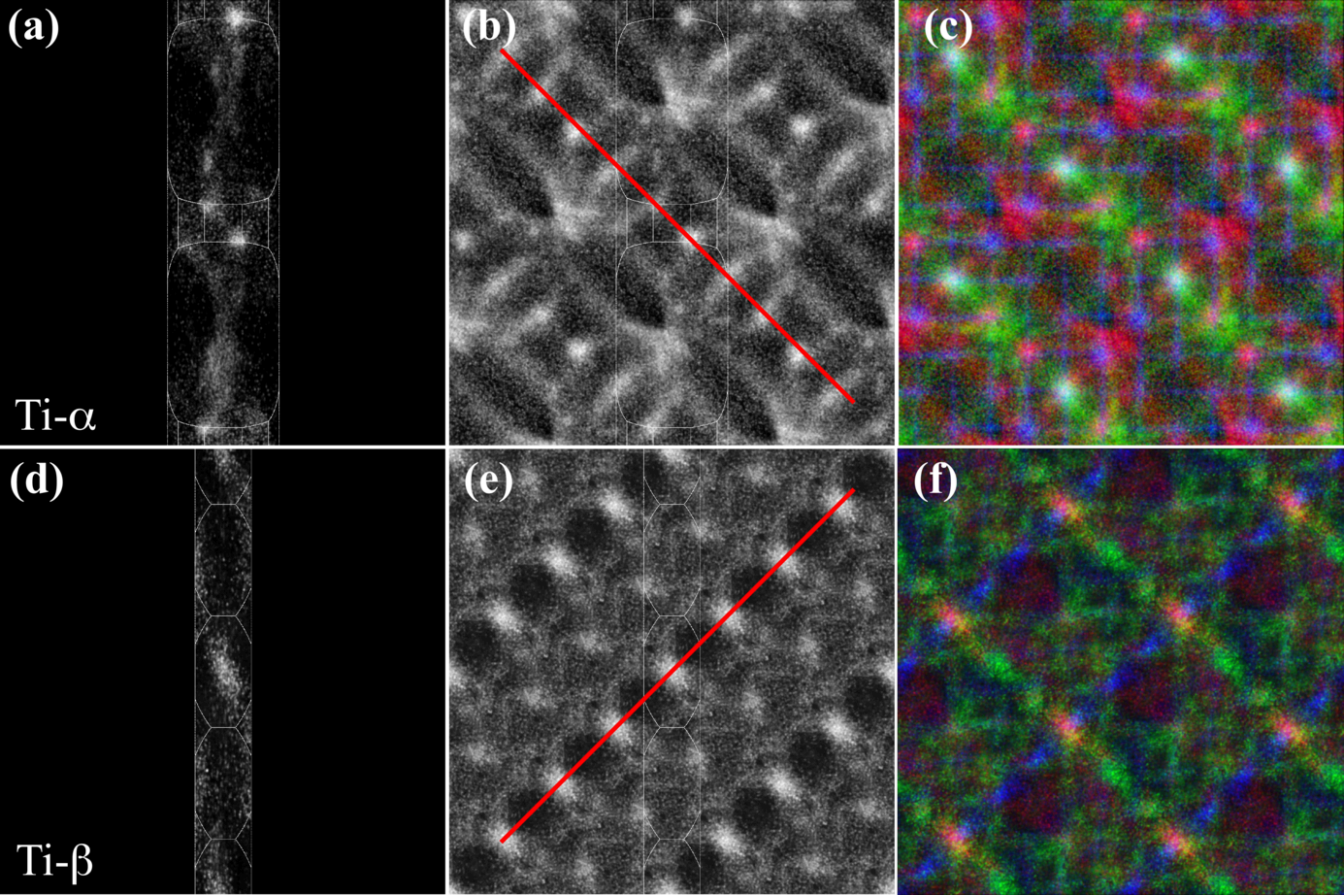
Ti-α (top row) and Ti-β (bottom row), (*a*) and (*d*) square torus representations using the respective fundamental zones, (*b*) and (*e*) symmetrized with (*q*, −*q*) double-cover equivalence and (*c*) and (*f*) RGB representations.

The second experimental example is based on a Ti–6Al–4V rolled-plate textured sample containing both α (h.c.p.) and β (b.c.c.) phases (Callahan *et al.*, 2017*b* ▸). An EBSD scan of a 2.25 mm^2^ region with step size 0.5 µm acquired at 20 keV beam energy resulted in an orientation data set of 9 × 10^6^ sampling points. As reported by Callahan *et al.* (2017*b* ▸), the microstructure shows microtextured regions along the rolling direction with similar grain orientations, with a strong preference for [11.0] directions to be aligned along the sample normal direction. Fig. 11 ▸ shows the square torus RFZ representations for the α [panel (*a*)] and β [panel (*d*)] phases, as well as the fully symmetrized versions of the orientation data sets [panels (*b*) and (*e*)]. Note the two red lines superimposed on features of the maps; the lines are perpendicular to each other, which suggests that there may be a preferred orientation relation between the two phases. This is indeed the case and Callahan *et al.* (2017*b* ▸) showed that the two phases have the Burgers orientation relation between them. Figs. 11 ▸(*c*) and 11 ▸(*f*) show the RGB torus maps for the two phases.

### 4.4. Fiber textures

#### 4.4.1. F.c.c. fibers

Consider the α fiber in an f.c.c. material. Its orientations are located around the line (φ_1_, π/4, π/2) in Euler space, with φ_1_ ∈ [0, π/2]. The corresponding unit quaternions are obtained by setting

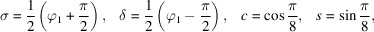

and then forming the quaternion as *q* = 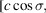 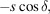 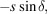 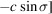 , resulting in

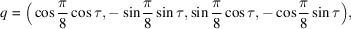

with τ = (2φ_1_ + π)/4. Conversion to the square torus coordinates (*X*, *Y*) using equation (4) results in 

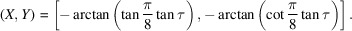

**Figure 12:**
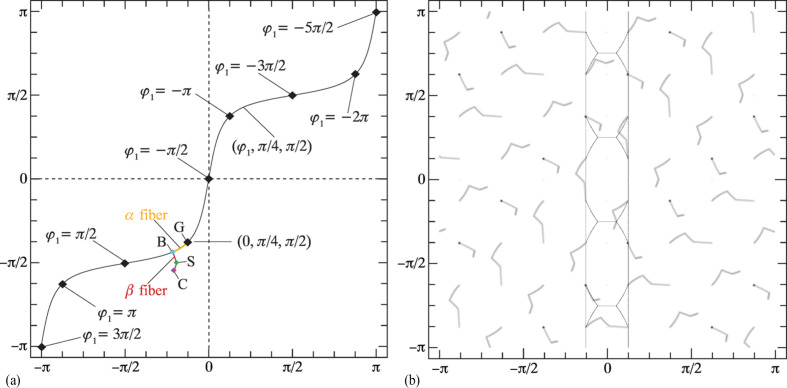
(*a*) A square torus representation of the line (φ_1_, π/4, π/2) in Euler space; the f.c.c. α fiber is highlighted in orange and the two segments of the β fiber in red. (*b*) In inverted contrast, all cubic symmetry operators are applied, as well as the (*q*, −*q*) double-cover property, for the α and β fibers. See text for additional explanation.

The square torus coordinates for the Goss point (0, 45°, 90°) are then (*X*, *Y*) = (−0.392699, −1.1781), and for the point where the β fiber branches off (φ_1_ = 35.26°) we have (*X*, *Y*) = (−0.674817, −1.35956); these points are indicated on the schematic diagram in Fig. 12 ▸(*a*).

For the β fiber, the S and C texture components are 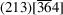 and 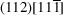 , respectively, leading to the square torus coordinates

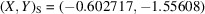

and

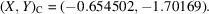

Fig. 12 ▸(*a*) shows these points, along with curved line segments corresponding to quaternion spherical linear interpolation (SLERP) between pairwise end points B–S and S–C. Note that the line (φ_1_, π/4, π/2) in Euler space maps onto the sigmoidal line from lower left to upper right shown in Fig. 12 ▸(*a*). Superimposed on the line are the locations for which φ_1_ is a multiple of π/2. Note that the range of φ_1_ is equivalent to [0, 4π], in agreement with the fact that the true periodicity of Euler space in the context of a quaternion mapping has all three Euler angles in the range [0, 4π] (Callahan *et al.*, 2017*b* ▸).

Application of the cubic symmetry elements and the (*q*, −*q*) double-cover property to the α and β fiber segments results in the square torus map shown in inverted contrast in Fig. 12 ▸(*b*); there are 48 equivalent locations for the fiber segments. Note that in some cases the α fiber maps onto a single point instead of a line segment.

#### 4.4.2. B.c.c. fibers

Consider the α, γ and ε fibers in a b.c.c. material. In Euler space, all orientations lie along the following lines: 

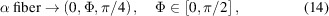




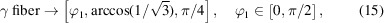




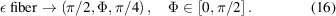




After conversion to the square torus coordinates, we find that the α fiber is represented by the curve

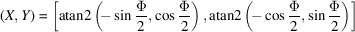

between the points (*X*, *Y*) = (0, −π/2) for Φ = 0 and (−π/4, −π/4) for Φ = π/2.

For the ε fiber we find

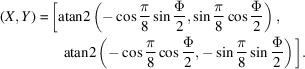

This curve intersects the *X* = 0 axis at the point (0, −π/2) and curves downwards towards the point 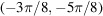 for Φ = π/2.

The γ fiber sits in between the two curves and is represented by

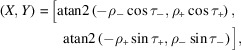

where 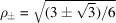 and τ_±_ = (π ± 4φ_1_)/8.

**Figure 13:**
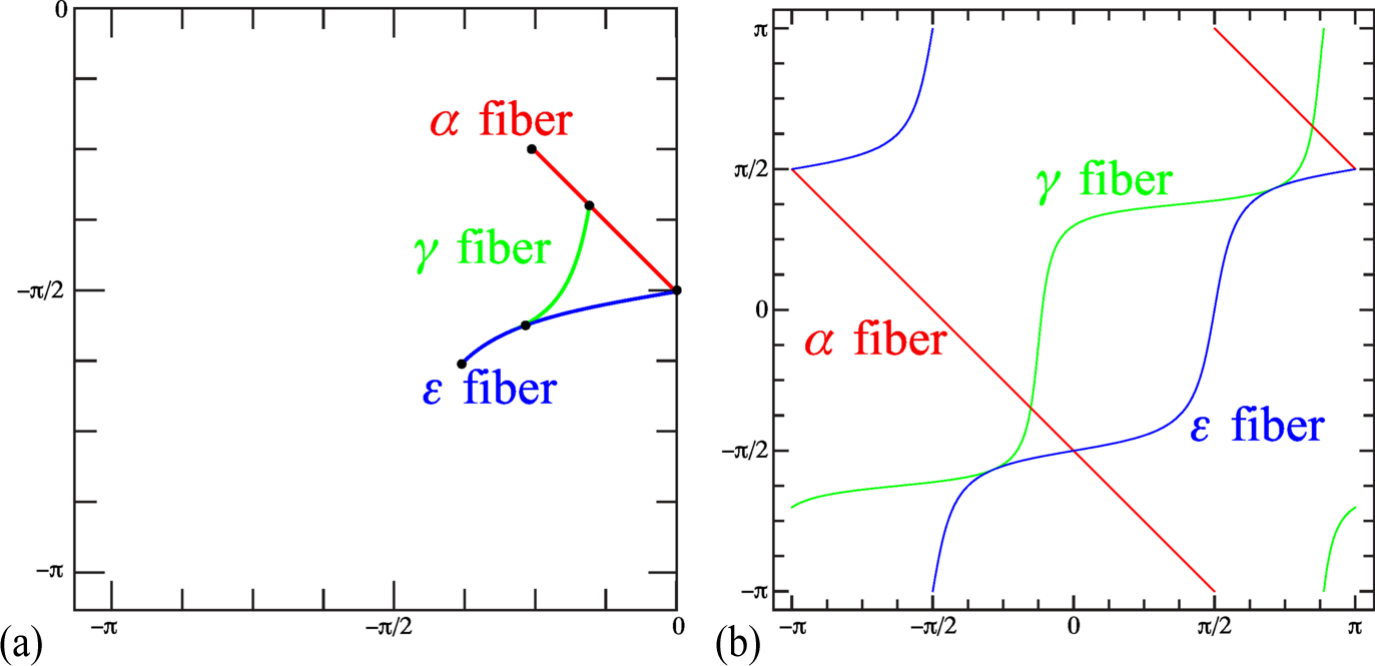
(*a*) A square torus representation of the α, γ and ε b.c.c. fiber textures in the lower left quadrant of the square torus map. (*b*) The full angular ranges of φ_1_ and Φ, as well as the (*q*, −*q*) double-cover property, are applied to the three fibers. See text for additional explanation.

The intersection points of the γ fiber with the α and ε fibers have coordinates 

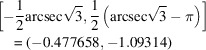

for the α fiber and 

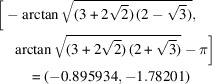

for the ε fiber. All the relevant points for the α, γ and ε b.c.c. fibers are shown in Fig. 13 ▸(*a*), which displays the lower left quadrant of the square torus map. The full square torus map for these b.c.c. fiber textures is shown in Fig. 13 ▸(*b*). In these plots, the free angle, φ_1_ or Φ, is swept through the entire range [0, 4π] to cover both *q* and −*q* quaternions.

### 4.5. Experimental fiber texture example

**Figure 14:**
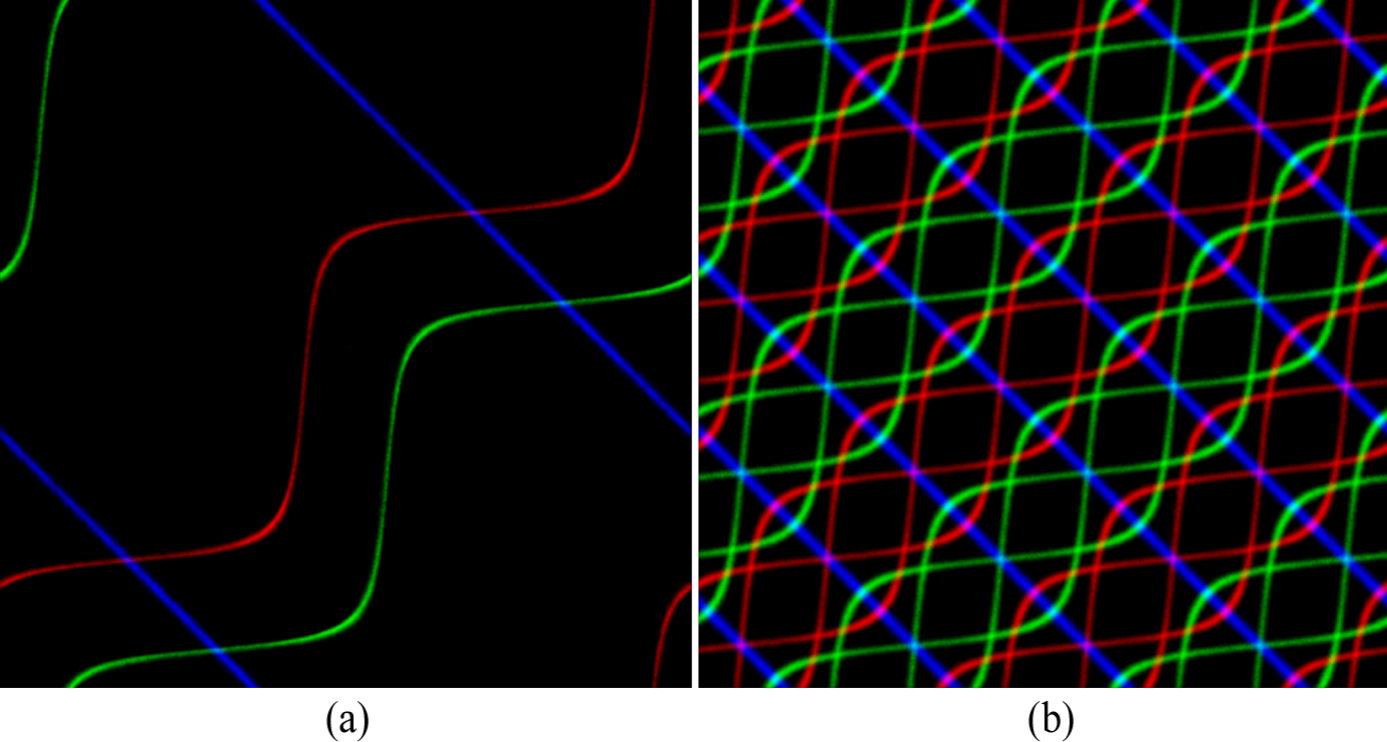
(*a*) An RGB square torus map representation of a strong γ fiber in an electrical steel [(*q*, −*q*) double-cover applied]. (*b*) All cubic rotational symmetry operators are applied to the experimental data. There are 28 306 data points in this data set.

As an example of a square torus representation of an experimental fiber texture, we consider a strong γ fiber in an electric steel; the data set consists of 28 306 orientations. When projected onto the (Φ, φ_2_) plane, the resulting distribution is a sharp nearly symmetric Gaussian with an FWHM of 2.1° around the point 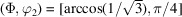 . Fig. 14 ▸(*a*) shows the RGB square torus map for this data set after application of the (*q*, −*q*) double-cover property; the intensity along the curves is nearly constant, in agreement with the nearly uniform distribution of the φ_1_ angle in the interval [0, 2π]. After application of the cubic rotational symmetry elements, the resulting RGB map is shown in Fig. 14 ▸(*b*).

## 5. Discussion and conclusions

In this paper, we have introduced a new 2D representation of material textures in terms of three isometric projections from the Clifford torus 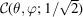 , a sub-manifold of the three-sphere 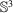 , onto square torus maps which are subsequently arranged in the red, green and blue channels of a periodic RGB image. Two of the maps are nonlinear projections involving the components of the Rodrigues–Frank vector. The third projection, which also involves Rodrigues–Frank vector components, was shown to be equivalent to a linear projection through Bunge Euler space along the Φ direction onto the (φ_1_, φ_2_) plane. Each 3D orientation, which has three degrees of freedom, is projected onto three periodic square torus maps and is represented in these maps by a narrow Gaussian peak; the superposition of all these peaks generates an intensity map that can optionally be modulated by a zone-plate function.

The zone-plate function representation is particularly useful to determine visually whether or not a set of orientations uniformly covers orientation space. For the orientation sampling algorithms of Section 3, the analysis in terms of the Riesz *s* energies provides some insight into the quality of the samples, *i.e.* how close to optimal the orientation sample is. For the Shoemake sampling, which has *r*_1_ and *r*_2_ values that are closer to unity than any of the other sampling methods, the value of *r*_3_, for *s* = 3, is nearly twice the optimal value. For increasing values of *s*, the Riesz energies become increasingly more sensitive to the local arrangements of sampling points, with only the nearest neighbors contributing in the limit *s* → ∞. For the orientation sampling 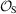 , the Riesz energies indicate that, on a global level, the sampling is very close to optimal, but at the local level there are some deviations from optimal. Whether or not these deviations are important depends on the application of the sampling. If the orientation set is to be used as an initial sampling for dictionary indexing, for instance (Singh & De Graef, 2016 ▸), then the local non-optimality is unimportant, since the indexing will typically be followed by an orientation refinement step where the orientations are allowed to wander away from the initial orientations.

Different from more conventional 3D representations of material textures, the RGB square torus map representation opens a unique path to the use of neural networks to automate the analysis of material textures, in particular to determine the mixture of texture components that are present in the orientation distribution. The use of ST maps in this context is the topic of ongoing investigations.

## Figures and Tables

**Table 1:** Riesz energy ratios for the five orientation sampling sets of Fig. 3 Each set contains 2 × 10^6^ samples (both *q* and −*q* are included in the Riesz summations). The first row shows the actual energies *E*_*s*_ for the optimal sampling. The ratios *r*_*s*_ are equal to unity for the optimal sampling.

Sampling	*r*_1_ (*E*_1_)	*r*_2_ (*E*_2_)	*r*_3_ (*E*_3_)
Optimal	3 395 305 452 627.101	4 000 000 000 000.000	12 315 331 182 477.914
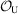	1.007 179 463 867	1.052 447 214 340	2.966 778 018 561
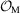	1.011 244 456 259	1.051 526 042 008	2.474 120 685 576
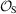	0.999 999 863 775	1.000 006 692 433	1.969 498 539 602
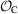	0.999 946 255 258	0.993 015 676 166	1.019 190 812 726
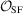	0.999 957 141 142	0.994 310 813 958	1.063 893 792 814
